# Supplementary material for: Double-duty caregivers enduring COVID-19 pandemic to endemic: “It’s just wearing me down”
Source: PLoS One. 2024 Apr 16;19(4):e0298584. doi: 10.1371/journal.pone.0298584 (PMC11020535; doi:10.1371/journal.pone.0298584)
Supplement: S1 File — (PDF) [file pone.0298584.s001.pdf]

Double-Duty Caregivers: Impacts of COVID-19

COVID-19 has Affected Family Caregivers. How have Canadian caregivers who are employed in healthcare and family caregivers (double-duty caregivers) been affected?

Dr. Jasneet Parmar and her research team from the University of Alberta are inviting family caregivers who are also working in healthcare to participate in a survey to understand the impacts of the dual caregiving roles.

Principal Investigator: Dr. Jasneet Parmar

Phone: 780-901-6236 jasneet.parmar@albertahealthservices.ca

Affiliations: Department of Family Medicine, Faculty of Medicine and Dentistry, University of Alberta, (Edmonton, Alberta)

Funding: Northern Alberta Academic Family Medicine Fund

Background. The COVID-19 pandemic has had a profound effect on all people in Canada, including family caregivers and healthcare providers. We expect that the impact may be even greater for “double-duty” caregivers. Double-duty caregivers are family caregivers who are employed in the healthcare field while also providing unpaid care outside of work (to a family member, chosen family, friend, or neighbor). The impact of the dual formal care role in healthcare and family caregiving can have implications for health providers. The purpose of this project is to examine the impact of COVID-19 on double-duty caregivers' wellbeing.

Why are you being asked to take part in this survey?

You are both a family caregiver and a health provider working in the healthcare system in Canada.

Study Purpose. We hope to understand how the COVID-19 pandemic and double-duty caregiving has impacted your caregiving and work experiences and what strategies you utilized to manage your personal and professional responsibilities, as well as your suggestions for supports going forward. By sharing your experiences and opinions, you will help Dr. Jasneet Parmar and her research team at the University of Alberta plan better supports for family caregivers during this COVID-19 pandemic and beyond.

How many people will participate in the survey? According to Statistics Canada data, there are 7.8 million family caregivers in Canada. Since 70% of family caregivers are employed, about 70% of healthcare providers may also be family caregivers. However, we do not know how many double-duty caregivers will participate in the survey given their busy lives, but we estimate it could range between 100 and 1000 people.

What will happen if I participate in this survey? We are inviting double-duty caregivers to complete this voluntary survey. The survey should take you approximately 15 to 20 minutes depending on how you respond to questions. Your responses will remain anonymous. No personal or identifying information is being collected and all data will be analyzed as a group (aggregated).

At the end of the survey, you will be asked whether you would like to be contacted for a follow-up interview about the impact of COVID-19 on you as a double-duty caregiver. You can participate in this survey regardless of whether you choose to be part of the follow-up study.

What are the potential risks or discomforts? Some questions that ask about how COVID-19 and double-duty caregiving have affected you may make you feel personally uncomfortable. Please note that you do not have to answer any questions you do not wish to answer or make you feel uncomfortable.

What are the potential benefits? There are no direct benefits to survey participants. However, the results of the survey may influence services to better support family caregivers. Results will be shared with family caregivers, the healthcare system, government, and caregiver organizations to strengthen their programs and assist with advocacy.

Do I have to participate? Completing the survey is your choice. You have the option not to answer any questions and still continue the survey. You can exit the survey at any time by closing the survey. Because we are not collecting personally identifying information, we cannot remove the answers once you have completed the survey.

Will my information be kept confidential? Your responses to this survey are completely confidential. It will not be possible to identify you in any report. All data will be kept confidential and stored securely. We are using a secure online platform called REDCAP. Researcher access to the survey is password-protected and transmission is encrypted. Survey responses cannot be linked to any identifying information (e.g., your name or email).

How long will the information be kept? In accordance with the University of Alberta data retention policy, we will keep the data for five years after the completion of the study (2027).

Questions and Concerns:

If you have any questions about this survey, please contact Dr. Sharon Anderson at 780-953- 5541 or sdanders@ualberta.ca

This study has received approval from the University of Alberta Health Ethics Research Board Study Number Pro00117700. If you have questions regarding your rights as a study participant, please call the University of Alberta Research Ethics Office at reoffice@ualberta.ca.

Submission of the survey implies your consent to participate.

---

1. Are you a health care provider? By health care providers we mean any individual employed in health occupations who are working in healthcare industries.

Your role is directly or indirectly related to the maintenance of the health of another individual.

You could be a physician; nurse; EMTs/paramedics; psychologist, social worker, allied health provider (audiologists, respiratory, speech-language, occupational, radiation, and physical therapists, diagnostic medical personnel, imaging specialists; nutritionists/dietitians); assistants (therapy, medical, and physician); recreation therapists; health care aides; personal care attendants; exercise science professionals (exercise physiologists, kinesiotherapists); health educators (asthma educators, diabetes educators); counselors (genetic counselors, mental health counselors, family therapists); specialists in cancer diagnosis and treatment; pharmacists and pharmacy personnel (pharmacy technicians and assistants).

◯ Yes, I am employed as a healthcare provider.
◯ Yes, I am a healthcare provider, but I am not currently employed in healthcare.
◯ Don't know/ Not sure
◯ No, I am not a healthcare provider

2. What is your healthcare profession?

- ◯ Activity assistant
- ◯ Audiologist
- ◯ Diagnostic imaging specialist
- ◯ Dietitian
- ◯ Dietetic Aide
- ◯ Educators (asthma, diabetes);
- ◯ Emergency medical technician
- ◯ Exercise physiologist
- ◯ Family therapist
- ◯ Genetic counselor
- ◯ Health care aide
- ◯ Kinesiologist
- ◯ Medical assistant
- ◯ Mental health counselor
- ◯ Nurse, Geriatric
- ◯ Nurse, Licensed Practical nurse
- ◯ Nurse, nurse practitioner
- ◯ Nurse, Psychiatric
- ◯ Nurse, Registered
- ◯ Nursing assistant
- ◯ Nutritionist
- ◯ Occupational therapist
- ◯ Paramedic
- ◯ Personal care attendant
- ◯ Pharmacist
- ◯ Pharmacy assistant
- ◯ Pharmacy technician
- ◯ Physical therapists,
- ◯ Physician
- ◯ Physician assistant
- ◯ Psychologist
- ◯ Radiation therapist
- ◯ Recreation therapist
- ◯ Respiratory therapist
- ◯ Social work assistant
- ◯ Social worker
- ◯ Speech-language therapist
- ◯ Therapy assistant
- ◯ Other

2a, You checked another profession. Can you tell us your healthcare profession?

____

3. Are you a family caregiver? We define family caregiver (carer, care partner) as any person who takes on a generally unpaid caring role providing emotional, physical, or practical support in response to another person's disability, mental illness, drug or alcohol dependency, chronic condition, dementia, terminal or serious illness, frailty, or COVID-19.

- ◯ Yes, I am caring for a family member, chosen family, friend, or neighbor now.
- ◯ I have not provided this support in the past 12 months
- ◯ No, I am not providing care.
- ◯ Don't know/ Not sure

4. What province do you live in?

◯ Alberta
◯ British Columbia
◯ Manitoba
◯ New Brunswick
◯ Newfoundland and Labrador
◯ Northwest Territories
◯ Nova Scotia
◯ Nunavut
◯ Ontario
◯ Prince Edward Island
◯ Quebec
◯ Saskatchewan
◯ Yukon

First, we have some questions about Double Duty Caregiving. This Double Duty Caregiving Scale reflects your experiences of being a double-duty caregiver, a health care provider who simultaneously provides care to patients and to a person needing care.

A. EXPECTATIONS

The following are statements related to the expectations that health care providers may experience while caring for a family member, chosen family, friend, or neighbor. There are many factors that influence expectations of care, one of which may be that the family caregiver has a professional health care background.

Please indicate on a scale of 1-5 where 1 is 'strongly disagree' and 5 is 'strongly agree' how much you agree or disagree with each statement.

	Strongly Disagree	Disagree	Neutral	Agree	Strongly Agree
1. Because of my health care background, I have high expectations of myself to provide care to my family member, chosen family, friend, or neighbor.	○	○	○	○	○
2. Because of my health care background, I personally feel obliged to use my professional knowledge and skills to help care for my family member, chosen family, friend, or neighbor.	○	○	○	○	○
3. Because of my health care background, my care-receiver expects me to provide care for him/her.	○	○	○	○	○
4. Because of my health care background, my family expects me to provide professional care to my family member, chosen family, friend, or neighbor.	○	○	○	○	○

B. SUPPORTS
The following are statements related to the supports that health care providers may experience while caring for a family member, chosen family, friend, or neighbor.

"As a caregiver for a family member, chosen family, friend, or neighbor":
Please indicate how much you agree or disagree with each statement.

	Strongly Disagree	Disagree	Neutral	Agree	Strongly Agree
1. I can count on my friends and/or family to be there for me.	○	○	○	○	○
2. I receive help from my friends and/or family when I ask for it.	○	○	○	○	○
3. I can ask my colleagues for support in the care of my family member, chosen family, friend, or neighbor.	○	○	○	○	○
4. My workplace supports me to provide care to my family member, chosen family, friend, or neighbor.	○	○	○	○	○

C. KNOWING LIMITS
The following are statements related to knowing limits that health care providers may use to help them in providing care to their family member, chosen family, friend, or neighbor.

"Because I am a health care provider who also provides care to my family member, chosen family, friend, or neighbor" Please indicate how much you agree or disagree with each statement below.

	Strongly Disagree	Disagree	Neutral	Agree	Strongly Agree
1. I have clear-cut ideas of what I can do and what I can't do in caring for my family member, chosen family, friend, or neighbor.	○	○	○	○	○
2. I know my limits when I provide care to my family member, chosen family, friend, or neighbor.	○	○	○	○	○

D. SETTING LIMITS

The following are statements related to setting limits that health care providers may use to help them in providing care to their family member, chosen family, friend, or neighbor.

"Because I am a health care provider who also provides care to my family member, chosen family, friend, or neighbor" Please indicate how much you agree or disagree with each statement below.

	Strongly Disagree	Disagree	Neutral	Agree	Strongly Agree
1. I say "no" to requests to provide professional care for the person I care for FROM FAMILY MEMBERS.	◯	◯	◯	◯	◯
2. I say "no" to requests to provide professional care for the person I care for FROM OTHER HEALTH PROFESSIONALS .	◯	◯	◯	◯	◯

E. MAKING CONNECTIONS

The following are statements related to making connections that health care providers may use to help them provide care to their relatives.

"Because I am a health care provider who also provides care to my family member, chosen family, friend, or neighbor" Please indicate how much you agree or disagree with each statement below.

	Strongly Disagree	Disagree	Neutral	Agree	Strongly Agree
1. I use my professional connections to obtain quality care for my family member, chosen family, friend, or neighbor.	◯	◯	◯	◯	◯
2. I use my professional connections to access health care information for my family member, chosen family, friend, or neighbor.	◯	◯	◯	◯	◯
3. I tap into my professional resources to ensure quality care for my family member, chosen family, friend, or neighbor.	◯	◯	◯	◯	◯
4. I use my knowledge of the health care system to access the appropriate care for my family member, chosen family, friend, or neighbor.	◯	◯	◯	◯	◯

F. CAREGIVING INTERFACE
The following are statements related to the boundaries between professional care and family caregiving.

"Because I am a health care provider who also provides care to my family member, chosen family, friend, or neighbor" Please indicate how much you agree or disagree with each statement below.

	Strongly Disagree	Disagree	Neutral	Agree	Strongly Agree
1. I can never get away from providing care.	○	○	○	○	○
2. I feel pulled in two or more directions.	○	○	○	○	○
3. I feel like I am caught between two worlds (professional and personal).	○	○	○	○	○
4. I feel my professional care to my family member, chosen family, friend, or neighbor goes unrecognized.	○	○	○	○	○
5. I experience stress when my family caregiver and professional roles blur.	○	○	○	○	○
6. I struggle to keep my "health professional" and "family caregiver" roles separate.	○	○	○	○	○

Health and Wellbeing

**1. COVID-19 and double-duty caregiving may affect stress and anxiety.
Think about how you feel today. For each statement, please indicate how much you agree with the statement,**

	Not at all	Somewhat	Moderately	Very Much.
1. I am comfortable	◯	◯	◯	◯
2. I am anguished	◯	◯	◯	◯
3. I feel at ease	◯	◯	◯	◯
4. I feel nervous	◯	◯	◯	◯
5. I feel concerned	◯	◯	◯	◯
6. I feel good	◯	◯	◯	◯

2. In general, would you say your health is?

◯ poor
◯ fair
◯ good
◯ very good
◯ excellent

3. Over the last year, my PHYSICAL health has,

◯ Has improved
◯ Remained about the same
◯ Has deteriorated e.g., less fit, more pain, new illness)
◯ Don't know/ Prefer not to answer

4. Over the past year, my MENTAL health has

◯ Has improved
◯ Remained about the same
◯ Has deteriorated (e.g., anxiety, stress, worry)
◯ Don't know/ Prefer not to answer

5. COVID-19 may have affected your social relationships and networks. For each statement, please tell us how much you agree with the statements with Yes, More or Less, or No.

	Yes	More or Less	No.
1, I often feel rejected	○	○	○
2. I experience a general sense of emptiness	○	○	○
3. I miss having people around me	○	○	○
4. There are plenty of people I can rely on when I have problems	○	○	○
5. There are many people I could trust completely	○	○	○
6. There are enough people I feel close to	○	○	○

6. We would like to know how YOU are managing overall. Which of the following statements describes how you can manage right now? Select one answer.

- ○ I am active, energetic, and exercise regularly.
- ○ I am well, but only occasionally active. I manage finances/transportation/heavy housework on my own.
- ○ My health conditions are well managed, but I am generally inactive. I may require advice on how to obtain supports with finances/transportation/heavy housework.
- ○ I am more tired than I used to be, and have more troubles obtaining supports than before, but still can coordinate things myself.
- ○ I need physical or practical assistance with finances, transportation, or heavy housework.
- ○ I need assistance with out-of-home activities, struggle with stairs, and could use help with my bathing or medications.
- ○ I need help with all my personal care
- ○ I am completely dependent for all personal care (dressing, eating, help to go to the bathroom).
- ○ I am very ill and near the end of my life.

Demographics

1. In which healthcare setting are you primarily employed?	◯ primary care<br>◯ acute care<br>◯ home care<br>◯ supportive/assisted living<br>◯ long-term care<br>◯ emergency medical care<br>◯ community or social care<br>◯ Not currently employed as healthcare provider<br>◯ Other
2. How old are you?	◯ 16 to 24<br>◯ 25 to 34<br>◯ 35 to 44<br>◯ 45 to 54<br>◯ 55 to 64<br>◯ 65 to 74<br>◯ 75 or older<br>◯ Prefer not to answer
3. What is your gender? Which of the following do you identify with?<br>Please select one answer.	◯ Woman<br>◯ Man<br>◯ Transgender<br>◯ Non-binary<br>◯ Other
4. Do you live in an urban, suburban, rural or remote setting?	◯ Urban<br>◯ Suburban<br>◯ Rural<br>◯ Remote
5. What is your marital status?	◯ married<br>◯ living common-law<br>◯ widowed<br>◯ separated<br>◯ divorced<br>◯ single, never married<br>◯ Don't know/Prefer not to answer
6. What is the highest level of education you have completed or the highest degree you have received?	◯ Less than high school diploma<br>◯ High school diploma or equivalent (e.g., GED)<br>◯ Some college not completed<br>◯ Certificate<br>◯ Diploma<br>◯ Bachelor degree<br>◯ Graduate degree<br>◯ Prefer not to answer

7. Please choose your ethnicity:	◯ Black<br>◯ Caucasian/ White<br>◯ Filipino<br>◯ First Nations<br>◯ Hispanic or Latinx<br>◯ Inuit<br>◯ Metis<br>◯ South Asian<br>◯ Southeast Asian<br>◯ West Asian<br>◯ Other<br>◯ Prefer not to answer
7a, You selected "other" ethnicity. Please tell us what ethnicity we missed	__________________________________

Employment

1. In an average week, how many hours are you working in your healthcare employment?	◯ 0 hours/week<br>◯ 1-14 hours/week<br>◯ 15-29 hours/week<br>◯ 30-34 hours/week<br>◯ 35-39 hours/week<br>◯ 40 hours/week<br>◯ 41-49 hours/week<br>◯ 50 or more hours/week<br>◯ Prefer not to answer/Don't know
2. Has your employment status changed as a result of your caregiving?	◯ Yes<br>◯ No<br>◯ Prefer not to answer/ don't know
2a You said yes, your employment changed because of your caregiving. How has it changed? Please select the reason that best applies,	◯ Working more hours due to caregiving<br>◯ Working fewer hours due to caregiving<br>◯ On a leave of absence due to caregiving<br>◯ Left of employment due to caregiving<br>◯ Laid off temporarily due to caregiving<br>◯ Working from home due to caregiving<br>◯ Don't know/ Prefer not to answer<br>◯ Other
2b. You selected other reasons your employment changed because of your caregiving. Please tell us how employment changed.	______________________________
3. Is there anything you would like to tell us about the impact of your family caregiving on your employment during the COVID-19 pandemic? The impacts could be positive, negative, or both. Did COVID-19 play a role?	______________________________
4. Is there anything you would like to tell us about the impact of your employment on your family caregiving during the pandemic? The impacts could be positive, negative, or both. Did COVID-19 play a role?	______________________________

Family Caregiving Role

1. In an average week, how much time do you spend providing care to a family member, chosen family, friend, or neighbor ? There are 168 hours in a week.	◯ 1 hour a week or less<br>◯ 2 to 9 hours<br>◯ 10 hours<br>◯ 11 to 20 hours<br>◯ 21 to 30 hours<br>◯ 31 to 40 hours<br>◯ 41 to 80 hours<br>◯ 81 to 120 hours<br>◯ 121 to 168 hours<br>◯ Prefer not to answer/ Don't know
2. During the past 12 months, how many family members, friends, or neighbours have helped you with your family caregiving?	__________________________
3. How many years have you been a family caregiver providing care for a family member, chosen family, friend, or neighbor? (Please round your answer to a whole number of years e.g.,1, 2 3, 6, 10)	__________________________
4. In the last year during the COVID-19 pandemic, have you experienced financial hardship because of your caregiving responsibilities? Please select one answer.	◯ No financial hardships because of caregiving responsibilities.<br>◯ A few financial hardships because of my caregiving responsibilities.<br>◯ Moderate financial hardships because of my caregiving responsibilities.<br>◯ Yes, a lot of financial hardships because of my caregiving responsibilities.
5. Are you a "sandwich generation caregiver", defined as caring for children under the age of 18 (your children, stepchildren, grandchildren) and are a family caregiver to a family member, chosen family, friend, or neighbor?	◯ Yes<br>◯ No<br>◯ Don't know/Prefer not to answer
5A In the last 12 months, which of the following parental care tasks have you primarily been responsible for? Check all that apply	☐ Taking the children to and from school or daycare centre<br>☐ Staying home with the children<br>☐ Homeschooling, supervising online schooling, or helping children with homework<br>☐ Putting the children to bed or seeing that they go to bed<br>☐ Playing with the children or taking part in leisure activities with them
6. How many people do you care for?	◯ 1 person<br>◯ 2 people<br>◯ 3 or more<br>◯ Prefer not to answer

About the family member, chosen family, friend, or neighbor you care for.
When answering the next set of questions please think about the care receiver you SPEND the MOST TIME caring for.

1) What is your relationship to the person you care for? Please select one answer

- ◯ Parent/In-Law
- ◯ Spouse/Partner
- ◯ Child
- ◯ Chosen family
- ◯ Sibling
- ◯ Other Relative
- ◯ Friend
- ◯ Neighbor
- ◯ Employer/ employee
- ◯ Prefer not to answer/ don't know

2) Where does the person you spend the most time caring for usually live? Please select one answer.

- ◯ They live with me, in the same household as I do.
- ◯ They live separately in their own private household (house, condo, apartment) or with another caregiver
- ◯ They live in supportive living (e.g., lodge, assisted living or supportive living)
- ◯ They live in long-term care or a group home
- ◯ Other

2a You answered that you care for someone in "another" living situation. Please tell us where they are living?

____

3) Please estimate how long it takes you to travel (one -way) by automobile to provide care for your family member, chosen family member, friend or neighbor.

- ◯ Live with me
- ◯ less than 10 min,
- ◯ 11-30 min,
- ◯ 31-59 min,
- ◯ 60 min (1 hour)
- ◯ 1 to 2 hours
- ◯ 3 to 6 hours
- ◯ 7 to 12 hours
- ◯ 13 to 24 hours
- ◯ More than 24 hours
- ◯ Don't know/ Prefer not to answer

4) How old is the person you SPEND THE MOST TIME CARING FOR? (e.g. Less than a year, 1, 2, 3, ... 70, 80, 100)

____

Your turn

1. We have asked you many questions, is there anything that you would like to tell us about double duty caregiving or what you think needs to be done to support double-duty caregivers? ____________________
