# Supplementary material for: Double-duty caregivers enduring COVID-19 pandemic to endemic: “It’s just wearing me down”
Source: PLoS One. 2024 Apr 16;19(4):e0298584. doi: 10.1371/journal.pone.0298584 (PMC11020535; doi:10.1371/journal.pone.0298584)
Supplement: S2 File — (DOCX) [file pone.0298584.s002.docx]

# S2: Double Duty Caregiving Scale Dimensions

## Comparison of Cronbach’s Alphas 2009 and 2022 Studies

| Subscale | Items | Number included | Cronbach’s Alpha Double-duty Caregivers, 2022 | Cronbach’s Alpha Validation Study, 2009 |
| --- | --- | --- | --- | --- |
| Expectations | 4 | 415 | .79 | .83 |
| Supports | 4 | 415 | .76 | .71 |
| Know limits | 2 | 415 | .84 | .67 |
| Set limits | 2 | 415 | .77 | .65 |
| Connections | 4 | 415 | .78 | .75 |
| Caregiving Interface | 6 | 415 | .81 | .81 |

## Comprehensive Explanations of the Six Dimensions of the Double Duty Caregiver Scale

**Expectations (4-items)** A total of 4-items were developed to address two proposed sources of ‘Expectations’ to provide care to an elderly relative: 1) *Professional Expectations* (2-items) and 2) *Familial Expectations* (2-items). Professional expectations may stem from health professionals who are involved directly in the care of the relative or from professional colleagues or friends who are not involved directly; professional expectations include those that may be held by DDCs themselves based on their credentials. Familial expectations may come directly from the elderly recipient of care, with his/her requests for assistance, or may come from other family members.

A subscale total for each proposed source of ‘Expectation’ is obtained by summing items 1, 2 for *Professional Expectations* and items 3,4 for *Familial Expectations*. A sum of both domains is then calculated for a total subscale score for ‘Expectations’. Scores range between 4 and 20.

Higher scores represent greater expectations to provide care.

# Supports (4-items) Four items were developed to assess ‘Supports’, reflecting the sources of support from: 1) Personal Supports (2-items) and Professional Supports (2-items) caregiving domains. Personal domains of support for DDCs usually include some degree of emotional, informational, and substantive support from family members and friends. Professional domains of support often include access to material, informational or emotional support from the DDC’s workplace or colleagues at work.

A subscale total for each source of ‘Support’ is obtained by summing items 1, 2 for *Personal Supports* and items 3, 4 for *Professional Supports*. A sum of both domains is then calculated for total subscale score for ‘Supports’. Scores range between 4 and 20. Higher scores represent greater supports to provide care.

# Knowing Limits (2-items) Two items were developed to assess ‘Knowing Limits’ (2-items). Because DDCs were constantly assessing their personal and professional boundaries of care, ‘Knowing Limits’ represents the extent to which DDCs are aware of their capacity to provide care.

A subscale total is obtained by summing both items. Scores range between 2 and 10. Higher scores represent greater awareness of limits to provide care.

# Setting Limits (2-items) Two items were developed to test ‘Setting Limits’ (2-items). This subscale refers to taking action in order to maintain boundaries between personal and professional care domains. Actions include refusing to provide hands-on (typically perceived as professional) care to their elderly relatives.

A subscale total is obtained by summing both items. Score range between 2 and 10. Higher scores represent greater action to set limits to provide care.

# Making Connections (4-items) Four items were developed to test ‘Making Connections’ (4-items). Making Connections examines the multiple interactions that serve to bring two domains closer together. These include accessing healthcare information or care for one’s relative, using professional knowledge and connections to obtain care, or navigating the healthcare system. A subscale total is obtained by summing all four items. Scores range between 4 and 20. Higher scores represent greater making connections to provide care.

# Caregiving Interface (6-items) Six items were developed to measure the caregiving interface (CI), or the degree of blurring between the professional and personal caregiving domains (intermediate domain). Two sources of CI were established: 1) *Perceptions* and 2) *Consequences*. The *Perceptions* relate to how the DDC perceives their ability to maintain boundaries of care, this dimension addresses the extent of the connections between elder caregiving and other forms of gendered caring work (e.g. nursing). The *Consequences* reflect the effects of the perceived blurring of boundaries and encompasses both feelings about the blurring of roles and the psychological impact of these feelings.

A subscale total for each proposed source of Caregiving Interface is obtained by summing items 1, 2, 3 for *Perceptions* and items 4, 5, 6 for *Consequences*. A sum of both sources is then calculated for total subscale score for ‘Caregiving Interface’. Score range between 6 and 30.

Higher scores represent greater blurring of professional and personal caregiving boundaries.
