# Supplementary material for: Double-duty caregivers enduring COVID-19 pandemic to endemic: “It’s just wearing me down”
Source: PLoS One. 2024 Apr 16;19(4):e0298584. doi: 10.1371/journal.pone.0298584 (PMC11020535; doi:10.1371/journal.pone.0298584)
Supplement: S3 File — (DOCX) [file pone.0298584.s003.docx]

# S3: Individual Interview Semi-Structured Interview Guide

Note re interview methodology: We will adjust the interview guide as we learn how research participants make sense of their experiences and we make analytic sense of their meanings and actions.

## Introduction:

Thank you for taking part in this interview.

My name is [and something about yourself] The study team is led by Dr. from the University of Alberta. She is a Care of the Elderly Physician, so she understands healthcare and works with family caregivers. She has also been a family caregiver to her dad and now to her mother.

Overview:

From this stage of the research, we wish to learn about how the COVID-19 pandemic and double-duty caregiving has impacted your caregiving and work experiences and what strategies you utilized to manage your personal and professional responsibilities, as well as your suggestions for support going forward.)

We sent you Information about the study and the Ethics Consent form, I want to get your verbal consent to the questions,

| Do you understand that you have been asked to participate in a research study? | Yes □ No□ |
| --- | --- |
| Have read the information above? | Yes □ No□ |
| Do you understand the risks and benefits involved in taking part in this research study? | Yes □ No□ |
| Do you understand that the discussions will be recorded? | Yes □ No□ |
| Do you understand that you have an opportunity to ask questions and discuss this study? | Yes □ No□ |
| Do you know how to contact the researchers if you want to ask questions and discuss this study? | Yes □ No□ |
| Do you understand that you are free to withdraw from the study as outlined above, without having to give a reason and without penalty? | Yes □ No□ |
| Do you understand the issue of confidentiality and anonymity outlined above? | Yes □ No□ |
| Do you understand who will have access to the information you provide? | Yes □ No□ |

Do you have any questions?

We realize you are busy, and we appreciate your time. We expect the interview will take about 45 to 60 minutes.

Anonymity:

We would like to recording the interview. (If on ZOOM) You can turn off your camera if you would like. I am going to delete the video recording immediately after the interview. The digital audio recordings will be kept in an encrypted folder on a password-protected University of Alberta computer safely in a locked facility. They will be transcribed word for word.

May I record the discussion to facilitate its recollection? (If yes, switch on the recorder).

Despite being recorded, I would like to assure you in any reports or academic articles, you will not be identified. Any information that would allow you or your work setting to be identified will be removed from the transcriptions.

## Guiding questions for participants:

## Icebreaker question

1. First, can you tell me a little about your family caregiving?
2. Now can you tell me a bit about your current role and work setting?

## Continuing questions

1. Has your caregiver role changed since COVID-19 began?”

Probe: Can you tell me a bit about the changes? You mentioned….. I am curious…

1. Has COVID-19 and family caregiving impacted your work experiences?”

Probe: Can you tell me a bit about the impacts? I am curious about what your think might have helped.

1. Has COVID-19 and being employed in healthcare impacted your family caregiving experiences?”

Probes: Can you tell me a bit about those interactions between your work and care work at home.

How your colleagues/ other health providers have supported you as a family caregiver.

I am curious, have any healthcare providers you see with your (care recipient) or for your own health asked you how you are as a family caregiver?

I sent you a graphic of the three pro-typical experiences of double-duty caregivers before the COVID-19 pandemic. Double-duty caregivers tend to shift between making it work, working to manage, and living on the edge depending on work, family caregiving and other life experiences.

Making it Work: Most of the time caregivers in this prototype experience the benefits of caregiving, expectations are managed well and the level of resources to assist are high.


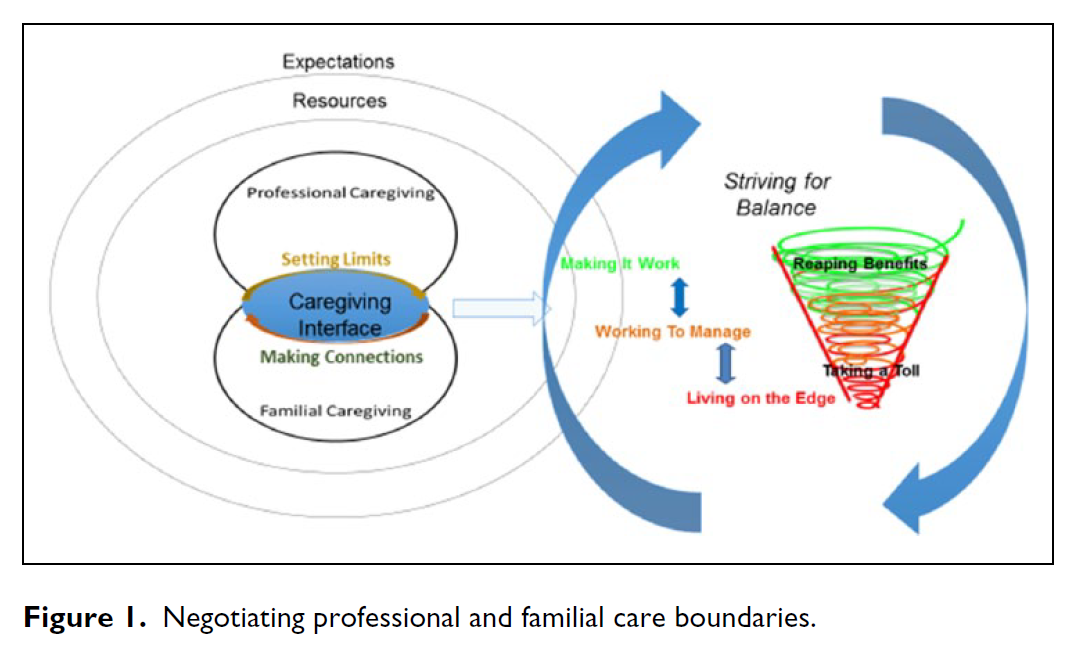
Working to Manage: Caregivers in this prototype experience some benefits of caregiving, their expectations are moderate and resources to assist them are limited.

Living on the Edge: Caregivers in this prototype experience high expectations that are not well managed and have weak resources to assist with the demands.

These protypes are very changeable, you might be living on the edge during a health or family crisis, and then move to making it work or working to manage as the crisis subsides. We are interested in your experiences in COVID

1. When you are thinking about your experience which of the three, making it work, working to manage, or living on the edge is closest to your experience right now?

Probe: Thinking about during the throes COVID-19, which of the three might have best reflected your experience?

Probe What about now, when everyone is tired from COVID? What has happened to your experience of managing work and family caregiving?

1. Are there benefits you are experiencing as a double-duty caregiver? Can you tell me about those?
2. Now think about how we can make family caregiving and work in healthcare work or manageable: “What strategies/ policies would you like to see implemented to help you manage that personal family caregiving and professional care responsibilities?”

Probe: What supports would assist you to better manage your care and your health and wellbeing?

Final question:

1. We have asked you lots of questions, is there anything that you would like to tell us?

End of interview:

Thank you for participating. You have provided us with very thoughtful information. If there is anything else, you would like to tell us please email us.
